# Persistent long-term habitat use by Florida manatees at Fort Pierce, Florida from 1997 to 2020

**DOI:** 10.1371/journal.pone.0297636

**Published:** 2024-03-21

**Authors:** Rachel Tennant, Beth Brady, Kim Love, Eric Ramos, Ryan Schloesser

**Affiliations:** 1 Manatee Observation and Education Center, Fort Pierce, Florida, United States of America; 2 Mote Marine Laboratory, Sarasota, Florida, United States of America; 3 K. R. Love Quantitative Consulting and Collaboration, Athens, Georgia, United States of America; 4 Fundación Internacional para la Naturaleza y la Sustentabilidad, Chetumal, Quintana Roo, México; 5 University of Vermont, Burlington, Vermont, United States of America; The City College of New York, UNITED STATES

## Abstract

To survive cold winters, Florida manatees (*Trichechus manatus latirostris*) depend on artificial (i.e., power plants) and natural warm water sources such as springs and passive thermal basins. Passive thermal basins can provide critical habitat for manatees for short or extended periods of time. The Henry D. King Powerplant in Fort Pierce, Florida discharged warm water into Moore’s Creek until it went offline in 1995. However, it is unknown to what degree manatees continue to occupy this area and how environmental factors influence their occurrence in the creek. To explore this, we examined the habitat use of Florida manatees in Moore’s Creek after the shutdown from November 1997 to March 2020 from daily counts of manatees. In addition, we correlated local environmental data (ambient air, temperature, salinity) to assess if Moore’s Creek had properties indicative of a passive thermal basin. Results indicated there was not an increase or decrease in habitat use over twenty years in the Creek. The consistent use of Moore’s Creek over the study period suggests that this habitat possesses thermal and freshwater resources to support manatee occurrence long-term. These findings provide robust support for the importance of this habitat and passive thermal basins for Florida manatees.

## Introduction

The Florida manatee (*Trichechus manatus latirostris*) is a subspecies of the West Indian manatee (*T*. *manatus*) distributed across the southeastern United States [1]. During the non-winter months (approximately April to October), Florida manatees are primarily observed in waters as far north as Georgia [2] and west to Alabama [3]. Sirenia are tropical, herbivorous mammals and as such, have little need for thick blubber for insulation. Additionally, due to their low metabolic rate [4, 5], manatees have a limited tolerance to cold water. During the winter months (approximately November to March), the manatee population is concentrated in Florida. But even in Florida, water temperatures can drop below 17°C and sometimes as low as 10°C for several days [4, 6]. Prolonged exposure to cold temperatures can cause cold stress syndrome resulting in hypercoagulability of the blood [7]. This in turn can compromise manatee immune systems, leading to increased susceptibility to pathogenic infections, lesions, pneumonia, impaired myocardial function, and potentially death [5, 7–9]. Because of their limited tolerance to cold, manatees are commonly observed at warm water refugia during the winter months [10].

Warm water refuges for Florida manatees include natural springs, warm-water discharge sites from power plants, and passive thermal basins (PTB) [10, 11]. The majority of manatees seek shelter in power plant effluents or artesian springs where water temperatures consistently remain between 20–22°C [12]. Passive thermal basins are waters where cooling processes are slow and temporarily retain pockets of relatively warm water [13]. PTB are dynamic in their ability to retain warm water temperatures due to changes in the environment (i.e., solar radiation, convection, groundwater seeps, weather conditions) [11, 14]. In turn, these sites can provide thermal shelter for manatees during short or extended periods of cold weather [14, 15]. For example, Ten Thousand Islands is a critical PTB which was thought to be effective in preventing mass mortality from cold stress for manatees in southwestern Florida during the severe winter of 2009–2010 [12]. Additionally, several other habitats inhabited by manatees like canals, stormwater drainage ditches, dredged basins, sewage outfalls, and creeks are slightly warmer than surrounding waters and may serve similar functions to PTB [10, 11].

There is growing concern about the removal of artificial warm water refuges for manatees and the subsequent challenge of finding new sources [10, 11]. Manatees exhibit strong site fidelity, returning annually to specific individual warm-water refuges or clusters of such refuges [2, 16, 17]. Previous studies have shown that when primary warm water sources were temporarily unavailable, manatees persisted in visiting the site regularly [15, 18, 19]. Few studies have observed manatees remaining in close proximity to the primary refuge, utilizing PTB during colder periods [2, 19]. As a result, understanding how Florida manatees use alternative warm water sources, such as PTB, is vital for their conservation and the safeguarding of their critical habitats.

The Henry D. King Power Plant in Fort Pierce, Florida, was once a thermal refuge for Florida manatees in the Indian River Lagoon. The warm water discharged by the plant was directed into Moore’s Creek, where manatees would gather during the winter months. The power plant went offline in 1995 and was decommissioned in 2008. It is unclear how the loss of this warm water refuge has affected the presence of manatees in the area. To gain insight into this question, we used a unique dataset of long-term manatee counts at Moore’s Creek gathered by trained volunteers over 23 years. Our analysis aimed to determine if manatee use of the creek has changed over time and identify the environmental factors driving these changes. This allowed us to gain a deeper understanding of the importance of the creek as a habitat for manatees and the potential impacts of the loss of the power plant as a thermal refuge.

## Methods

### Study location

**Fig 1:**
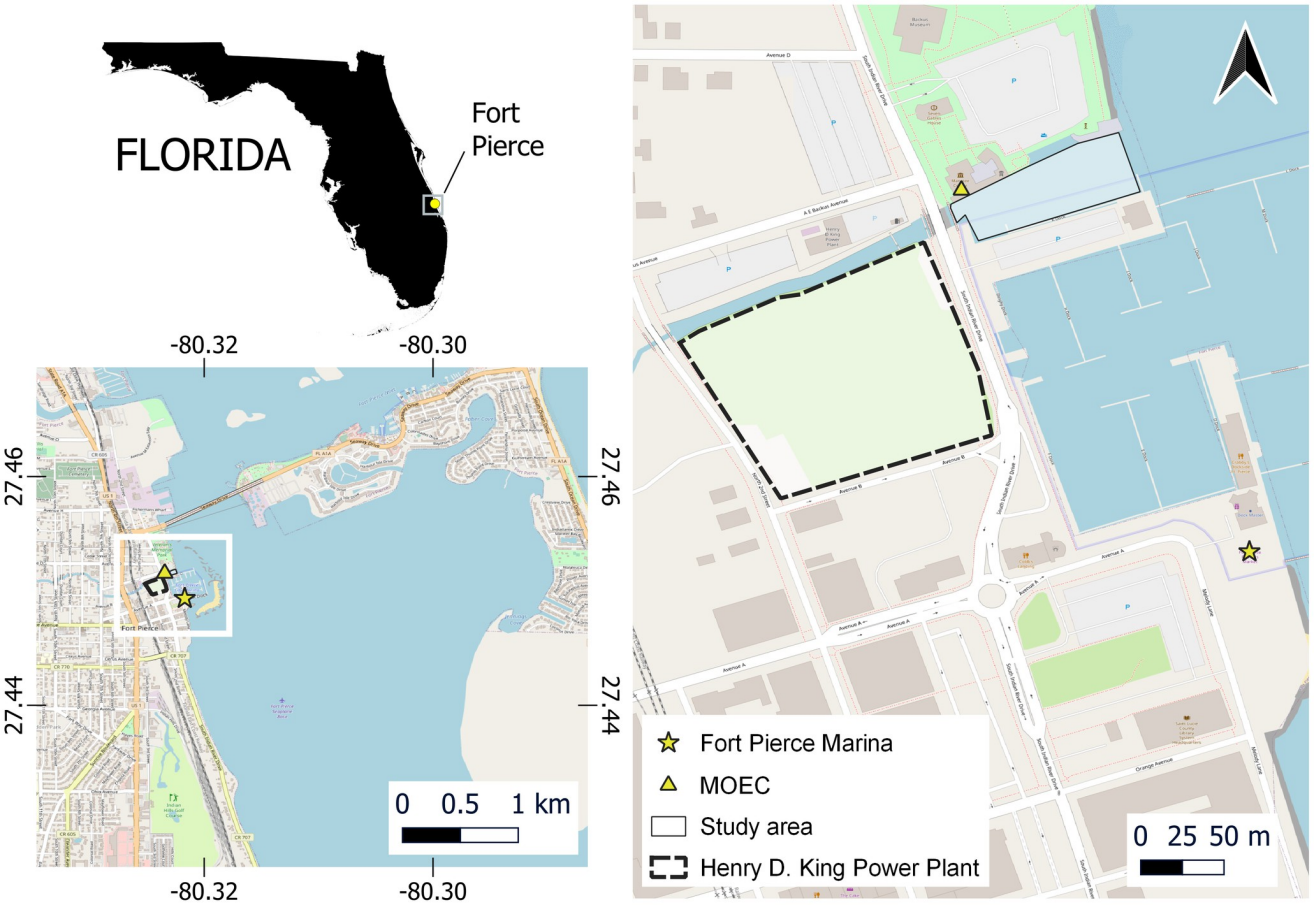
Map of the study site at Moore’s Creek in Fort Pierce, Florida, USA. The white box in the inset map highlights the location of the Fort Pierce Marina (yellow star), the Manatee Education and Observation Center (MOEC) (yellow triangle), the study area (thin black outline), and the former Henry D. King Power Plant (dashed black outline). The basemap was acquired from OpenStreetMap (https://www.openstreetmap.org/copyright) and is licensed as open access under the Creative Commons Attribution-ShareAlike 2.0 license.

Data on manatee activity for this study were collected at the Manatee Observation and Education Center (MOEC) at Moore’s Creek (27.4516° N, 80.3235° W) in Fort Pierce, Florida, USA. The creek has an average depth of 1 m, a substrate of mud and silt, and flows directly into the Indian River Lagoon. The creek is partially enclosed by a seawall that extends 185 m behind the center, 109 m in front of the center, and ranges from 31–39 m in width (Fig 1).

### Manatee count

MOEC volunteers record manatee sightings in Moore’s Creek and have been collecting this data since 1996. Volunteers receive a basic training that lasts four hours followed by an additional 4–8 hours of shadowing a more experienced volunteer. They are also provided supplementary training on spotting sick/injured manatees. Volunteers are scheduled for 3.5 hr shifts (10:00–13:30 h), and 13:30–17:00 h) six days per week during full-season hours (October to June) and at least three days per week during summer hours (July to September). Volunteer hours vary based on volunteer availability and are tracked in Volgistics software [20].

Volunteers count the number of manatees spotted each day. Manatees were identified in the following ways: observations of the snout or the back at the surface, a fluke print, or visualization of the full body beneath the surface [21]. The number of manatees seen each hour were counted during the time that the Center is open. The official manatee count recorded for the day was the highest number written in a single hour on that day (i.e. the largest cluster seen that day). This method may lead to under-reporting the number of manatees spotted in Moore’s Creek, but ensures that manatees are not inadvertently double-counted.

Observations were collected from December 1996 to December 2020. In 1996, the power plant was still online generating warm water outflow for manatees. Since the plant was offline in subsequent years, we excluded the year 1996 from analysis. Observations collected from 1997 to 2020 were the most consistent from November through March. Data collected from April through October from these years was either not recorded consistently, no observations were collected or the center was closed. For this reason, this analysis focused on the most reliable data obtained during the months of November to March which is when manatees aggregate at warm water refuges in Florida.

Data for this study was collected through passive observations from land on the property of the Manatee Observation and Education Center. It did not require ethical approval from an institutional review board or research permitting.

### Statistical analysis of manatee counts

Summary statistics were calculated for the number of manatees observed over time. In order to determine whether the presence of manatees over time was generally increasing or decreasing in a statistically significant trend, a generalized estimating equation (GEE) model was used. This model assumes the manatee counts are distributed according to a negative binomial model, which is often considered appropriate for count data. The GEE model also allows incorporation of a correlation across observations. Each season of observations from November to March was considered a “cluster” of observations in which the observations were correlated over time. The specific correlation structure chosen for this analysis was the autoregressive correlation structure where there is a correlation between observations that follow one another directly in time order (AR(1)). This is appropriate for manatee counts, since the number of manatees that appear on one day is more likely to be related to the number of manatees that appear on nearby days than those many days in the future or in the past. It is also appropriate that this occurs within individual seasons, as the correlations should not carry from the end of one season to the next. Fixed effects in the model include year and month. The observations are allowed to be correlated within each season using the AR(1) correlation structure. The negative binomial distribution also includes a scale parameter that allows estimation of variance in accordance with the negative binomial distribution. Note that “year” in this model is defined as the study year, where year 0 is November 1997 to March 1998, year 1 is November 1998 to March 1999, etc. Year 23 only includes November and December of 2020. GEE models were conducted using SAS [22].

### Environmental data

We investigated the relationship between air temperature, water temperature, and salinity to determine if Moore’s Creek had environmental characteristics that support manatee usage. Moore’s Creek is part of a 2,832 acre drainage basin that discharges stormwater into the creek. Prior studies indicate manatees require fresh water for physiological needs (osmoregulation [23]) and the freshwater runoff from storm drainage may draw manatees to Moore’s Creek. Ambient air temperatures were investigated, as sustained and sharp drops in temperature influence water temperatures and in turn increase manatee occurrence at warm water sites [24]. Average daily ambient air temperatures at the St. Lucie County International Airport (27.2942° N, 80.2205° W) in Fort Pierce were obtained from the National Oceanic and Atmospheric Association’s National Weather Service (www.weather.gov). The St. Lucie International Airport is 7.2 km inland from the MOEC.

An ORCA Kilroy Unit was installed at the mouth of Moore’s Creek in 2018. Every thirty minutes, temperature and salinity levels were measured. To determine if Moore’s Creek was warmer and fresher than the surrounding area, we obtained temperature and salinity data from a land /ocean biogeochemical observatory (LOBO; Harbor Branch Oceanographic Institute) unit deployed in Fort Pierce. The Fort Pierce water quality station is located in the Indian River Lagoon (27.4756° N, 80.3266° W). It is situated north of the Fort Pierce North Causeway Bridge (close to the Fort Pierce Inlet), approximately 2.4 km from the MOEC.

Hourly temperature and salinity data were extracted from archived LOBO data (http://fau-hboi.loboviz.com/). Data from the Kilroy, LOBO and ambient air data from February 20, 2018 to January 25, 2020 were analyzed. Water temperature and salinity data were averaged within dates (November to March) to associate with daily counts of manatees. Mean daily water temperatures were also used to calculate differences in water conditions within and outside the creek for dates with manatee counts when environmental data was available from both sources.

The lag between low air temperatures and decrease in water temperature to less than 20°C can influence when manatees travel and occur at warm water outflows [24]. Therefore, the influence of environmental conditions with a lagged effect on manatee behavior were examined by first assessing pearson correlation coefficients between mean air temperature from the day manatee counts were obtained and 1–5 days prior to obtaining counts (using all days with data available). The final step was calculating the effective change in temperature over that time period for inclusion in further analyses.

The relationship between daily manatee counts and environmental conditions were examined with a generalized linear model (GzLM) assuming a negative binomial distribution of manatee counts. The full model included additive effects of the change in air temperature from the previous day (based on the greatest correlation from the lag analysis), water temperature in the creek, salinity, and the difference in water temperature within and outside the creek as predictors. The full model was compared to reduced models made up of all possible combinations of the aforementioned factors using Akaike Information Criterion (AIC), with the ‘best model’ determined by the lowest AIC value. The GzLMs were run using the glm.nb function in R statistical software [25].

Finally, because average ambient air temperatures from St. Lucie International Airport were available between November of 1999 to February of 2020, the GEE described in the previous section was extended to include an effect of ambient air temperature as well as an interaction of air temperature and month. This allows examination of the long-term relationships between air temperature and manatee count, as well as an examination of the relationship of air temperature and manatee count by month, as weather conditions vary.

## Results

### Manatee counts

**Fig 2:**
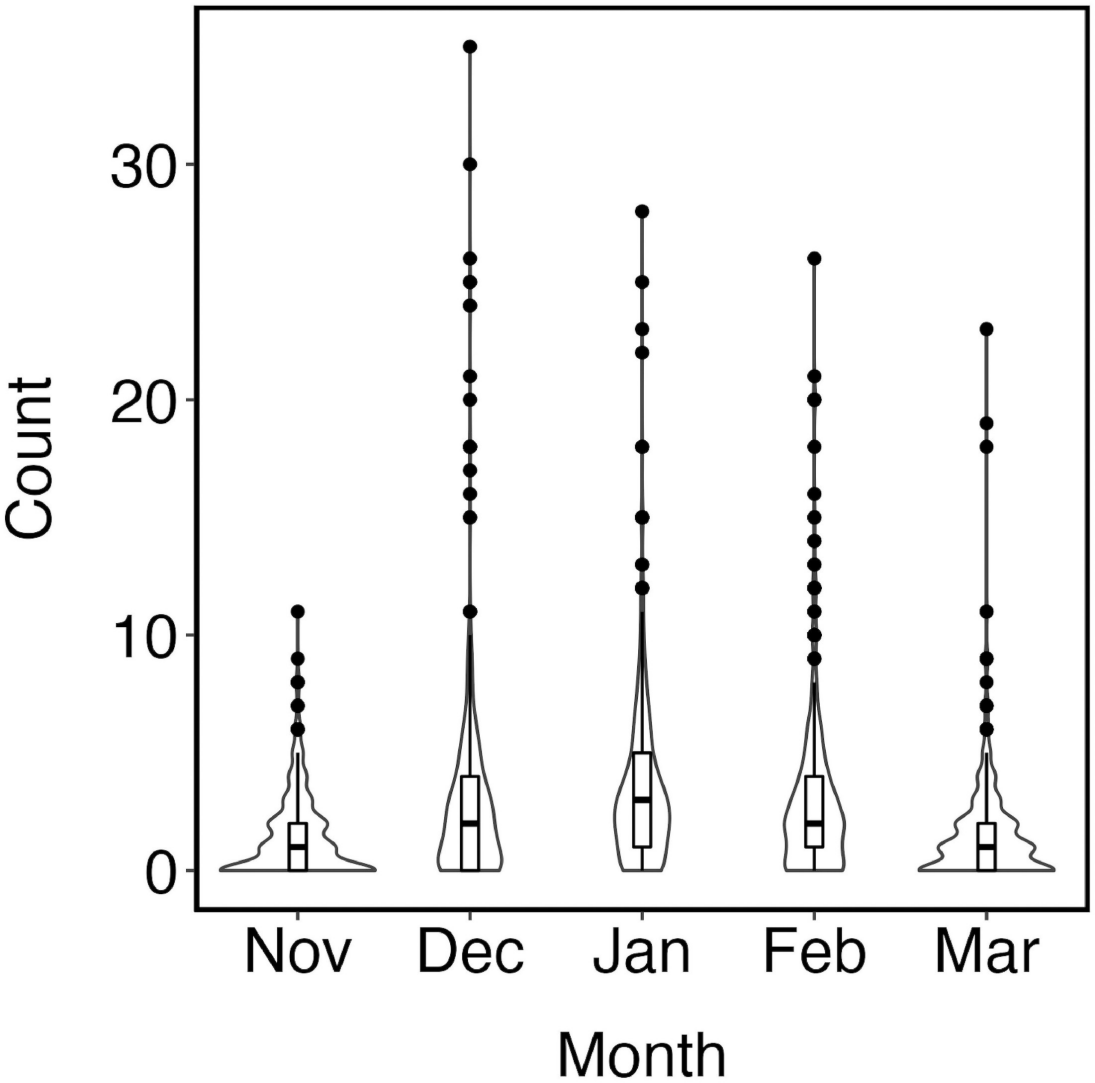
Violin plots depicting manatee counts by month from November to March, from 1999 to 2020. The black dots represent outliers. The black line in the boxplot represents the median and the upper and lower boundaries of the box represent the 75% and 25% quartiles respectively.

Approximately 8,345 hours of observations were spent observing manatee occurrence in Moore’s Creek from November 1997 to December 2020. Volgistics data indicated eight individual observers spent over 1,000 hours observing manatees with at least 30 individuals having spent over 100 hours observing manatees. Manatee observations resulted in 6,697 sightings over 2,923 days. The number of manatees observed on any given day ranged from 0 to 26 manatees with an average of 2.29 manatees (SD = 2.84) sighted each day. The highest manatee counts were observed in December and January (Fig 2).

### Manatee presence over years

The GEE model described in the statistical methods was used to investigate whether the average daily number of manatees encountered in Moore’s Creek changed over time. The model included all 2,923 observations. There was not a statistically significant effect of year in the model, although there was a statistically significant effect of month (Table 1). This means there was no evidence of an overall increasing or decreasing trend over time, although there was statistically significant seasonal variation from month to month.

**Table 1:** Overall tests of GEE model terms.

Variable	df	LR χ2	p
**Year**	1	0.07	0.7959
**Month**	4	17.71	.0014

**Fig 3:**
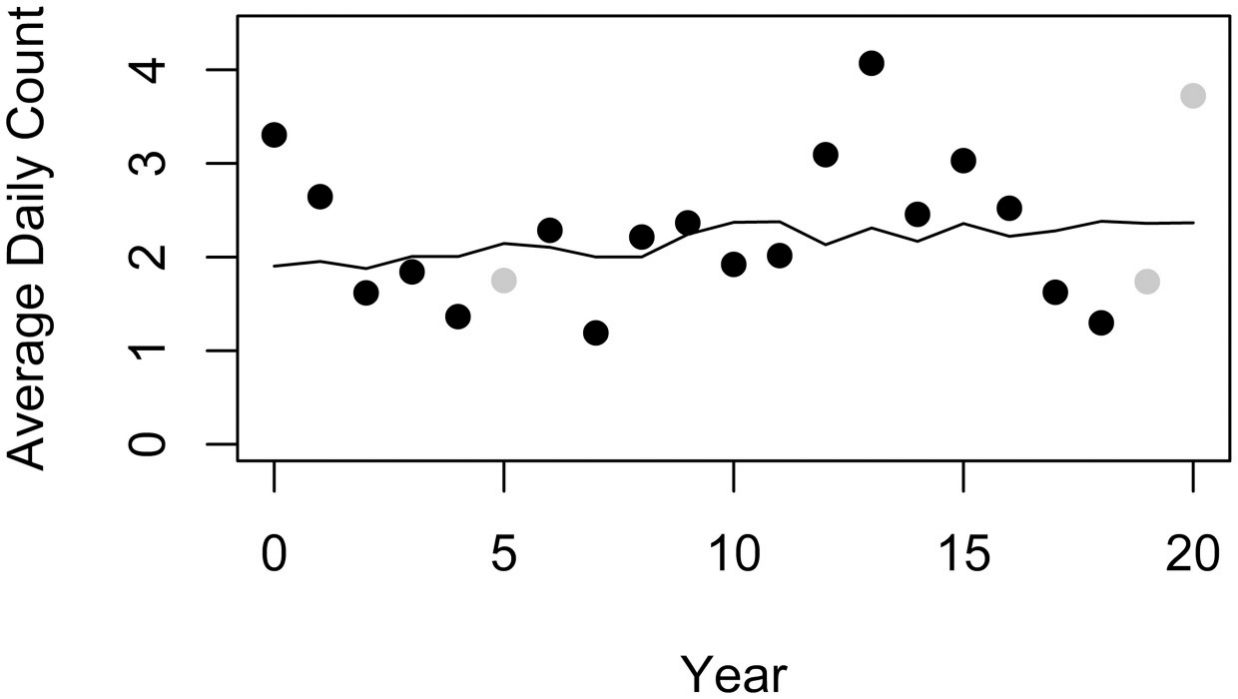
Scatterplot of data with the averaged data values (first averaged over days by month, and then averaged over the monthly averages by year) with the non-significant decreasing trend from the model superimposed. This demonstrates the fit of the model, as well as the small size of the yearly increasing trend relative to the year-to-year variability in the counts. Light gray points are from years that did not include data from all 5 months and would therefore not reflect the model estimates.

The estimated coefficient for year is -0.0027; this corresponds to a rate ratio of 0.997 (Table 2). This means that within the data set, there is a non-significant decreasing trend in the average daily manatee counts of 0.3% each year (Fig 3).

**Table 2:** Estimates of GEE model coefficients. December was used as a reference month in the model and is not reported here.

Parameter		Estimate	SE	RR	Z	p
Intercept		0.924	0.145	2.519	6.36	< 0.0001
Year		-0.003	0.011	0.997	-0.25	0.7987
Month	January	0.287	0.087	1.332	3.30	0.0010
Month	February	0.145	0.136	1.156	1.07	0.2847
Month	March	-0.481	0.126	0.618	-3.82	0.0001
Month	November	-0.615	0.112	0.541	-5.51	< 0.0001

### Environmental variables over years

**Fig 4:**
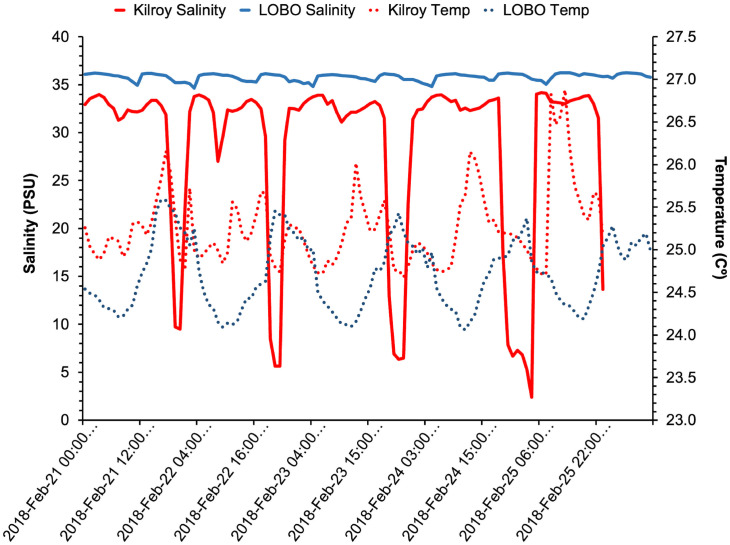
Line graph illustrating hourly and daily trends in salinity (PSU) and temperature (Cº) data recorded by the LOBO (blue) and the Kilroy (red) from February 21 to 25, 2023.

Temperatures in Moore’s Creek ranged from 6.94 to 29.17°C. When compared with the LOBO data, temperatures within Moore’s Creek were on average 0.42°C warmer than the LOBO location, with more extreme conditions resulting in temperatures as much as 2°C warmer or 1.9°C colder (Fig 4). Salinity in Moore’s Creek ranged from 0 to 36 ppt. The fluctuation in the creek corresponded with an outflow of freshwater (i.e., fresh salinities, 0–15 ppt) during low tides and an influx of seawater (higher salinities, 23–36 ppt) during high tides (Fig 4).

The potential for broader weather patterns to be reflected in habitat use of manatees was greatest using air temperature from 1 day prior based on the relationship between lagged air temperature, water temperature, and manatee counts (Table 3).

**Table 3:** Correlation between the daily mean air temperature data from 1–5 days prior to individual manatee counts, water temperature and manatee counts.

Lag in Air Temperature Data (# Days Prior)	Pearson Correlation with Water Temperature	Pearson Correlation with Manatee Counts
1	0.718	-0.225
2	0.598	-0.108
3	0.469	-0.097
4	0.411	-0.123
5	0.386	-0.056

The AIC scores from the GzLMs indicated that the best model describing manatee counts included water temperature and salinity within the creek, but did not include the effect of changes in air temperature from the previous day or the effect of differences in temperature within and outside the creek (S1 Table, with the results of the best model presented in Table 4). Manatee counts were highest when temperatures in the creek (Kilroy) were colder and the water was fresher in the creek.

**Table 4:** Results of the best model from the GzLM describing salinity and water temperature between Moore’s Creek and the LOBO data.

	Estimate	SE	Z	p
**(Intercept)**	4.074	0.773	5.270	<0.0001
**Salinity**	-0.029	0.012	-2.463	0.0138
**Water Temperature**	-0.106	0.030	-3.543	0.0004

**Fig 5:**
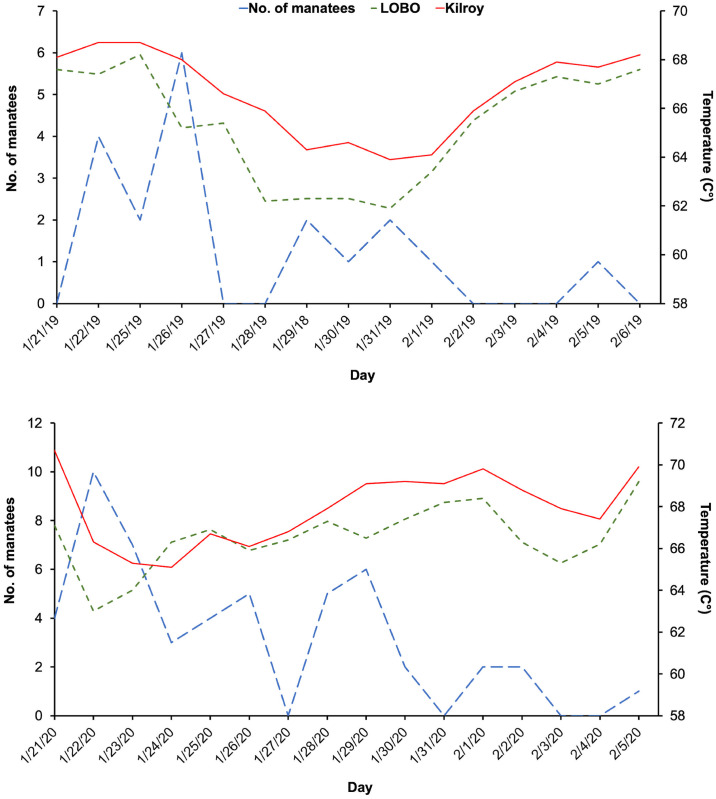
Manatee counts, LOBO temperatures, and temperature of Moore’s Creek (Kilroy) from January 21 to February 6, 2019 and January 21 to February 5, 2020. These dates correspond to LOBO water temperatures below 20°C.

To investigate water temperature and manatee occurrence, we compared temperatures for the LOBO and Kilroy for 25 days when temperatures outside the creek were lower than 20°C. Although temperatures inside the creek were not consistently warmer than outside the creek, manatees tended to use the creek in higher numbers when temperatures outside of the creek were lower than 20°C. Consistent with the statistical analysis for water temperatures, this trend lasted for a day or two, until water temperatures inside Moore’s creek were similar or lower than outside of the creek (Fig 5).

The long term effects of ambient air temperature were modeled using the GEE approach outlined in the methods section. The model included 2,584 observations. There was not a statistically significant effect of year in the model, despite a statistically significant interaction of month and ambient air temperature (Table 5). This means there was still no evidence of an overall increasing or decreasing trend over time, and the model shows that ambient air temperature was related to daily manatee counts. The associated model coefficients are given in Table 6.

**Table 5:** Overall tests of GEE model terms with ambient air temperature.

Variable	df	LR χ2	p
**Year**	1	1.060	0.3028
**Month**	4	13.03	.0112
**Ambient Air Temperature**	1	11.05	0.0009
**Month x Air Temperature**	4	12.92	0.1117

**Table 6:** Estimates of GEE model coefficients with ambient air temperature. December was used as a reference month in the model and is not reported here.

Parameter		Estimate	SE	RR	Z	p
Intercept		0.707	0.156	2.028	4.52	< 0.0001
Year		0.014	0.014	1.014	1.02	0.3087
Month	January	0.226	0.087	1.254	2.6	0.0092
Month	February	0.190	0.142	1.209	1.34	0.1806
Month	March	-0.405	0.107	0.667	-3.78	0.0002
Month	November	-0.377	0.107	0.686	-3.54	0.0004
AvgDayTemp		-0.031	0.007	0.969	-4.67	< .0001
AvgDayTemp x Month	January	0.021	0.008	1.021	2.71	0.0067
AvgDayTemp x Month	February	0.029	0.008	1.030	3.49	0.0005
AvgDayTemp x Month	March	0.008	0.010	1.008	0.82	0.4103
AvgDayTemp x Month	November	-0.031	0.009	0.970	-3.44	0.0006

Because the effect of average daily temperature varies by month, Table 7 shows the relationship of average daily temperature with manatee occurrence for each month. The effect of average daily temperature is statistically significant in November, December, and March, but not in the months that are generally the coldest (January and February).

**Table 7:** Estimates of relationship of average daily temperature with manatee occurrence by month.

Month	Estimate	SE	RR	χ^2^	p
November	-0.062	0.008	0.940	58.39	< 0.0001
December	-0.031	0.007	0.969	21.78	< 0.0001
January	-0.011	0.008	0.989	2.02	0.1557
February	-0.002	0.006	0.998	0.11	0.7372
March	-0.023	0.009	0.977	6.62	0.0101

## Discussion

Florida manatees rely on warm-water refuges to survive cold winter periods. In this study, we investigated the long-term habitat use of Florida manatees at Moore’s Creek, Fort Pierce, Florida from 1997 to 2020. Despite the decommissioning of the power plant, the number of manatees using the region has remained relatively constant over two decades. Further, the analysis indicates Moore’s Creek does not have properties of a thermal refuge that consistently maintains temperatures greater than 20°C. However, the creek is slightly warmer than outside the creek for a day or two when water temperatures drop below 20°C. This suggests that the creek has properties of a passive thermal basin by having warmer temperatures for short periods of time.

Our study correlated these observations with local environmental data (ambient air, temperature, salinity) to analyze how manatee presence fluctuated in the area in relation to oceanographic conditions. We discovered that Moore’s Creek exhibited temperature and freshwater properties that attracted manatees, particularly during winter months. Slightly warmer temperatures were observed in the creek compared to its surroundings, possibly due to radiation from the adjacent seawall, convection, less wind, and shallow depths [10]. Although salinity in the creek varied, manatee sightings increased when the waters were fresher, suggesting that they also sought freshwater at this location. Ambient air temperatures indicated manatee use of Moore’s Creek was highest during November, December, and March. Stronger and more sustained cold fronts occur during January and February, which could in turn lower water temperatures in the creek more quickly and result in fewer observations of manatees on a daily basis. Moore’s Creek may be used as a stopover point during November, December, and March as manatees travel to better quality warm water refuges. Regardless, the water temperatures at this site support its dual function as both a PTB and a freshwater source for manatees [10, 12].

Despite the shut down of the powerplant, manatees frequenting Moore’s Creek demonstrated consistent visitation patterns over a span of two decades. Although individual manatees could not be identified at this site, it is probable that the same individuals, as well as their offspring, returned to this location. Manatees exhibit site fidelity to various habitats that offer essential resources such as fresh water and warm water [26]. Along the Atlantic coast, radio-tagged manatees displayed strong site fidelity to specific warm-water refuges, consistently returning to the same ones [2, 16–18]. Similarly, tagged manatees were observed repeatedly traveling to the same freshwater sites for drinking purposes [15, 27]. Female manatees visiting Moore’s Creek may have imparted knowledge of this location to their calves. Telemetry studies suggest that independent calves exhibit similar site fidelity as their mothers [2, 16, 28].

It is unclear if other power plant locations would support manatee occurrence similar to Moore’s Creek if they shut down. Most power plants discharge directly into large rivers or bays. A potential scenario would be either in Fort Myers or Port Everglades power plants. Each of these sites discharge outflow into narrow canals. Canals have been used by manatees as a warm water source (i.e., DeSoto Canal, Satellite Beach). One study observed manatee occurrence at Port Everglades power plant when the plant went offline from 2013 to 2014. The study indicated that manatees used the area but in smaller numbers than observed in previous winters [29]. Further, they noted temperatures in some of the survey areas were slightly warmer and suggested this was due to exposure from direct sunlight and convection [29]. Further research would be needed to confirm how long temperatures remain above the critical threshold of 20°C in these locations.

The continuous utilization of this habitat over a twenty-year period underscores the significance of these sites for manatees. Our long-term citizen science data collection played a crucial role in identifying manatee presence in the area. This highlights the advantages of investing in the training of volunteers for dependable data collection, which can contribute to meaningful conservation efforts for threatened species. To gain a deeper understanding of individual site fidelity, ongoing monitoring could benefit from employing photo identification techniques using high-quality digital SLR cameras [30]. By leveraging advanced technology like drones [31], passive acoustic recordings [32, 33], and refining the data collection process, we can enhance our understanding of manatee behavior and habitat preferences, ultimately contributing to more effective conservation strategies and the protection of these vulnerable animals.

## Conclusions

This study highlights the enduring importance of Moore’s Creek in Fort Pierce, Florida, as a valuable habitat for the Florida manatee. The creek serves as a passive thermal basin for manatees as well as a source of freshwater. Our analysis reveals that even with the power plant closure, the number of manatees frequenting the area has remained consistent over the past two decades. These findings underscore the vital role of freshwater sources as well as current and former passive thermal basins as indispensable habitats for these marine mammals. Further research is necessary to investigate long-term trends in manatee habitat usage at sites affected by power plant closures, in order to determine the applicability of our findings to other regions within their range. Such research can contribute to a more comprehensive understanding of manatee conservation needs and inform future habitat protection efforts.

## Supporting information

S1 Table Akaike Information Criterion (AIC) scores from models examining the effect of various environmental factors on manatee counts, with the best model identified in bold font. X’s denote variables that are included in the models.
(DOCX)

S1 Data (XLSX)
