# Supplementary material for: Persistent long-term habitat use by Florida manatees at Fort Pierce, Florida from 1997 to 2020
Source: PLoS One. 2024 Mar 21;19(3):e0297636. doi: 10.1371/journal.pone.0297636 (PMC10956764; doi:10.1371/journal.pone.0297636)
Supplement: S1 Table — X’s denote variables that are included in the models. (DOCX) [file pone.0297636.s001.docx]

| **Model**  **Number** | **Salinity** | **Water**  **Temperature** | **Temperature**  **Difference** | **Air Temperature Change** | **AIC score** |
| --- | --- | --- | --- | --- | --- |
| 1 | X | X | X | X | 946.8940 |
| 2 |  | X | X | X | 952.9874 |
| 3 | X |  | X | X | 956.9032 |
| 4 | X | X |  | X | 945.5839 |
| 5 | X | X | X |  | 945.0851 |
| 6 |  |  | X | X | 962.0004 |
| 7 |  | X |  | X | 951.0877 |
| 8 |  | X | X |  | 951.2823 |
| 9 | X |  |  | X | 955.6349 |
| 10 | X |  | X |  | 954.9067 |
| **11** | **X** | **X** |  |  | **943.8242** |
| 12 | X |  |  |  | 953.6350 |
| 13 |  | X |  |  | 949.3678 |
| 14 |  |  | X |  | 960.0045 |
| 15 |  |  |  | X | 960.0488 |
